# Content validation of the Angioedema Quality of Life Questionnaire (AE-QoL) in a population of adult and adolescent patients with hereditary angioedema (HAE)

**DOI:** 10.1186/s41687-025-00876-3

**Published:** 2025-04-12

**Authors:** Lynne Broderick, April Foster, Laura Tesler Waldman, KD Jacobs, Laura Bordone, Aaron Yarlas

**Affiliations:** 1 QualityMetric, an IQVIA business, 1301 Atwood Avenue, Suite 216E, Johnston, RI 02919 USA; 2 Ionis Pharmaceuticals, Inc, 2855 Gazelle Court, Carlsbad, CA 92010 USA

**Keywords:** Hereditary angioedema, HAE, AE-QoL, HRQoL, Content validity

## Abstract

**Background:**

There is a lack of clear evidence pointing to a fit-for-purpose instrument to measure impacts on health-related quality of life (HRQoL) in adult and adolescent patients with hereditary angioedema (HAE). The purpose of this study was to determine whether the Angioedema Quality of Life Questionnaire (AE-QoL) is content valid and appropriate for capturing the impact of HAE attacks on HRQoL in both adults and adolescents with HAE.

**Methodology:**

This study used one-on-one, audio-recorded, cognitive debriefing interviews employing think-aloud and verbal probing approaches to evaluate the relevance, comprehensibility, and comprehensiveness of the AE-QoL in this patient population. All data were quality checked then coded and analyzed using inductive and deductive approaches.

**Results:**

This study included 10 adolescents and 12 adults with HAE in the United States. Overall, participants had positive impressions of the AE-QoL, finding the length and recall period appropriate, and the response options clear and easy to understand. Some reported minor concerns with the instructions, but none that prevented them from completing the instrument. Participants found the instrument relevant to their experiences with HAE, noting that items that were not personally relevant were still important to ask. Overall, participants found the AE-QoL comprehensible and comprehensive, although some participants, primarily adolescents, reported being unfamiliar with the word “leisure,” making it difficult to answer the item asking about impact of attacks on “leisure time.” Adolescents also reported that questions about school-related impacts were missing and would be important to ask about specifically.

**Conclusions:**

This study presents evidence that supports the content validity of the AE-QoL in adult and adolescent patients with HAE. While revisions could be considered prior to using the instrument with samples of adolescent patients with HAE, in general, adolescents and adults with HAE found the measure relevant, comprehensive, and comprehensible.

## Background

Hereditary angioedema (HAE), a rare, autosomal dominant disorder and a subtype of bradykinin-mediated angioedema (AE), is characterized by episodes of swelling that can be disabling, and are often unpredictable. These episodes, colloquially referred to as “HAE attacks,” can result in pain, disfigurement, and sometimes death, and have been shown to have impacts on health-related quality of life (HRQoL). HRQoL impacts in adults and adolescents with HAE include emotional distress, interference with daily activities and social functioning, and reduced work and/or school productivity [1–7]. Recent studies suggest the mean age of HAE symptom onset is between 10 and 12 years old [8, 9].

Advances in pharmaceutical development have resulted in long-term prophylactic and acute treatments for HAE, available for both adult and pediatric patients. Guidelines and recommendations for managing HAE [10, 11] note that in addition to changes in patients’ HAE symptoms, changes in their overall functioning, including impacts on HRQoL, must also be evaluated. However, clear evidence pointing to a fit-for-purpose, symptom-specific instrument to measure impacts of HAE attacks on HRQoL in adult and adolescent patients with HAE is lacking. The Angioedema Quality of Life Questionnaire (AE-QoL), a 17-item, patient-reported outcome (PRO) measure, was developed to assess the frequency of impacts of AE, or swelling, attacks on numerous aspects of patients’ everyday lives over the previous 4-week period [12]. The instrument measures HRQoL impacts across 4 domains (functioning, fatigue/mood, fear/shame, and nutrition), and categorizes the items as addressing “Areas of Daily Life” (5 items) and “Difficulties or Problems” (12 items). The AE-QoL does not capture symptoms, frequency or severity of AE attacks, nor the impacts having the disease itself has on HRQoL; rather, it was specifically developed and validated to assess the impacts of attacks on HRQoL in adult patients with recurrent AE (RAE). Respondents are asked to rate how frequently they experienced the impacts using a 5-point verbal rating scale (never, rarely, occasionally, often, very often).

The AE-QoL was developed for a broad patient population who experience RAE attacks, including, but not limited to, patients with HAE. It has previously been used to capture the impact of HAE attacks on patients’ HRQoL [3, 5, 6, 13] and has been psychometrically validated for use with adults [13–15]. Examples of the AE-QoL’s use include pivotal phase 3 clinical trials to measure benefit of long-term prophylactic treatments [5, 16–18]; however, evidence of its content validity in the published literature is limited to use with adults with HAE [1, 13, 14], and currently non-existent for use with adolescents with HAE.

The purpose of this study was to determine whether the AE-QoL is content valid and appropriate for capturing the impact of HAE attacks on HRQoL in adults and adolescents with HAE.

## Methods

This was a cross-sectional, non-interventional, qualitative, cognitive debriefing study. This study was conducted in accordance with industry and regulatory recommendations for best practices when evaluating the appropriateness of any clinical outcomes assessment selected for use with patients [19–21]. This study employed a purposive sampling strategy to recruit up to 12 adults and 12 adolescents with HAE. Purposive sampling is a non-probability sampling method commonly used in qualitative research studies [22]. This sampling strategy allowed the research team to use its knowledge of the research objectives and the population of interest to identify and include participants who would be appropriate for the study and help achieve the research objectives, including meeting specific sampling targets.

Recruitment efforts were conducted in partnership with a United States (US)-based patient advocacy group (PAG) focused on HAE. The PAG shared recruitment messaging with potential participants, or their parent/guardian. Interested individuals reached out directly to the PAG to be screened for eligibility. Prior to the start of recruitment efforts, all study materials were approved by an independent institutional review board (IRB; WCG Tracking #20224651). In an effort to protect the privacy of study participants, a waiver of document of consent was also approved by the IRB.

Individuals were eligible to participate in the study if they met the following criteria: ages 12–17 years for adolescents, or age ≥ 18 years for adults; had been told by a clinician that they have type I or type II HAE; had at least 1 HAE attack in the last 6 months; lived in the US; spoke and read English; and were able to provide confirmation of an HAE diagnosis.^1^ Individuals were excluded if they had HAE with normal C1-inhibitor.

The adolescent sample aimed to include 6 adolescents ages 12–14 years old, and 6 ages 15–17 years old, with one-half of each group identifying their sex as male and the other half as female. The adult sample aimed to include participants covering a broad range of ages (i.e., there were no sampling targets by age group for adults) and an even distribution between male and female participants.

Once an individual was deemed eligible to participate, a representative from the PAG reviewed the details of the study, including their rights as a study participant, with each participant (and their parent or guardian, in the case of adolescents). A study information sheet detailing this same information was then sent via email to each participant, or their parent/guardian in the case of adolescents.

Cognitive debriefing (CD) data were collected during 60-minute, one-on-one, audio recorded interviews. Each interview was conducted by a trained interviewer via web-conferencing software and followed a standardized, semi-structured interview guide developed to gather specific information related to the relevance, comprehensibility, and comprehensiveness of the AE-QoL.

Each interview began with a review of the study information sheet and a brief introduction to the study. Following this, all participants, including parents/legal guardians of adolescent participants were asked to provide verbal confirmation that they agreed to take part in the study. Next, participants were systematically asked questions about their experience with HAE, including current treatments, frequency of HAE attacks, and what it was like to have an attack. These data were used to capture participant characteristics to contextualize the study sample.

The interviewer then described the CD portion of the interview, during which participants debriefed a paper version of the AE-QoL (either printed out or onscreen). Participants were instructed in how to use a “think-aloud” method to read aloud all elements of the AE-QoL (instructions, questions, response options) and say what they were thinking as they read each element and answered the questions [20]. Upon completion of the think-aloud, the interviewers followed up with verbal-probing [20], asking participants targeted questions to evaluate the relevance, comprehensiveness, and comprehensibility of each element of the AE-QoL. Participants were asked to discuss any elements they may have noted as confusing and any instances where the interviewer noticed a pause or hesitation. At their discretion, parents/guardians of adolescent participants could remain present for their child’s interview. All parents/guardians who chose to remain present were reminded that the purpose of the interview was to gather input explicitly from their child and all questions were directed to the adolescent. Upon completion of the interview, each participant received a $100 honorarium in the form of a check distributed by the PAG.

Coding and analysis of the CD data was conducted using a 5-step process. First, upon completion of each interview, the interviewer populated a Microsoft Excel^®^ spreadsheet with interview data regarding any notable issues that arose (e.g., confusing or unclear items) or suggested changes to the AE-QoL (e.g., recommendations for improved wording). This spreadsheet tracked each of the issues or suggestions by participant, allowing the study team to easily identify issues or suggestions endorsed by individual and/or multiple participants. Each audio file was then shared, via a secure file transfer site, with an external transcription partner for verbatim transcription. All transcripts were reviewed by a member of the study team for inaudible content and accuracy before being accepted as final.

Second, as completed transcripts were received and quality checked, they were then cross-checked against the quick code spreadsheet. Cross-checking allowed for a full review of all data and confirmation that all feedback was accurately recorded during the quick coding process.

Third, transcripts were coded using NVivo qualitative analysis software (version 14.0, Lumivero, 2023) to identify information shared by participants, including overall opinions on the AE-QoL, and any other suggestions or insights. Coding captured whether suggestions were offered spontaneously or as a result of interviewer probing. All coding was completed by 2 members of the study team, who also conducted the interviews. The initial coding of 4 transcripts was reviewed during a coding consensus meeting during which the 2 coders identified and, through discussion, resolved any discrepancies or issues with coding or the coding structure. These meetings were held throughout coding as any issues or concerns arose, and all coding was reviewed by the study lead prior to analysis.

The coding was then used to evaluate the comprehensiveness, comprehensibility, and relevance of each aspect of the AE-QoL, confirming elements that were acceptable as-is and highlighting elements that were missing or might need to be revised. This information was ultimately used to determine what, if any, changes might be suggested.

Finally, suggestions to modify the AE-QoL were considered. The study team reviewed each of the issues identified during the interviews, noted the proportion and age group of study participants who raised the issue, and the nature of their feedback (e.g., whether the suggested edit was crucial to improving comprehension or simply a matter of personal preference). The study team evaluated each issue, including how consistently it was raised, and whether it was raised spontaneously or as a response to a probing question, and subsequently determined whether it was necessary to suggest a modification.

In addition, an item-tracking matrix was developed to track all elements of the AE-QoL in association with relevant comments that suggested a change, recommendations on whether and how to change, and the subsequent suggested edit(s), if appropriate [19].

## Results

### Participant characteristics

Ten adolescents^2^ and 12 adults with HAE took part in the CD interviews. 40% (*n* = 4) of the adolescent sample was 12–14 years old, and 60% (*n* = 6) was 15–17 years old (Table 1). The older adolescents were split evenly by sex (*n* = 3 male, *n* = 3 female), while most of the younger adolescent sample was female (*n* = 3, *n* = 1 male). The adult study sample had a median age of 41 years old (range: 20–65) and was split evenly by sex (*n* = 6 male, *n* = 6 female) (Table 2). Most participants (*n* = 7 adolescents, 70%; *n* = 11 adults, 92%) were taking long-term prophylactic treatment at the time of their interview and all were prescribed and using, when needed, an acute medication to treat HAE attacks. The adolescent sample experienced a median of 4.3 HAE attacks in the past 6 months and reported a range of 1–13 attacks, while the adult sample also experienced a median of 4.3 attacks in the last 6 months but reported a range of 1-100 + attacks.

**Table 1:** Adolescent cognitive debriefing participant characteristics

Adolescent participants (*N* = 10)		
		*n* (%)
**12–14 Years Old**		4 (40)
Male		1 (25)
Female		3 (75)
**Race/Ethnicity**		
Caucasian or White		3 (75)
Black		--
Hispanic or Latino		1 (25)
**15–17 Years Old**		6 (60)
Male		3 (50)
Female		3 (50)
**Race/Ethnicity**		
Caucasian or White		4 (67)
Black		--
Hispanic or Latino		2 (33)
**Treatment at the Time of Interview**		
Long-term prophylaxis	7 (70)	
Acute medication	10 (100)	
**# HAE Attacks in Past 6 Months**		
Median: 4.3; Range (min, max): 1, 13		

Abbreviation: HAE, Hereditary angioedema

**Table 2:** Adult cognitive debriefing participant characteristics

Adult participants (*N* = 12)	
**Age**	
Median: 41 years old; Range (min, max): 20, 65	
	**n (%)**
20–30 years old	3 (25)
31–50 years old	5 (42)
51+	4 (33)
**Sex**	
Male	6 (50)
Female	6 (50)
**Race/Ethnicity**	
Caucasian or White	8 (67)
Black	3 (25)
Hispanic or Latino	1 (8)
**Treatment at the Time of Interview**	
Long-term prophylaxis	11 (92)
Acute medication	12 (100)
**# HAE Attacks in Past 6 Months**	
Median: 4; Range (min, max): 1, 100+	

Abbreviation: HAE, Hereditary angioedema

### Overall impressions of the AE-QoL

Overall, across age groups, participants had positive impressions of the AE-QoL (*n* = 21; 95%). As one participant explained, “*everything is covered about HAE and…how it can affect your life*,* not…one topic [is] missed*” *(male*,* age 15).*

### Time to complete the AE-QoL

All participants (*n* = 22) judged the amount of time it took to complete the AE-QoL acceptable.

### Recall period

About three-quarters of the sample (*n* = 17; 77%) reported that the recall period (the last 4 weeks) was appropriate, and that it was easy to remember their attacks and the ways in which attacks impacted their lives over the last 4 weeks. Nevertheless, over half of participants (*n* = 14 total [*n* = 6 adults; *n* = 8 adolescents]; 64%) explained they would prefer a different recall period, some shorter and others longer (suggestions included the last 2 weeks; 3, 4, or 6 months; and “your last attack”) due to the frequency of their attacks and the variance in attack severity.

### Instructions

#### General

About three-quarters of the sample (*n* = 17; 77%) found the general instructions to be straightforward and clear. Among the other 5 participants, 2 adolescents (out of 10; 20%) were not familiar with the abbreviation “i.e.” used in the instructions; 1 adult (out of 12; 8%) found the instructions “too generic;” and 1 adult (8%) and 1 adolescent (10%) disliked the instruction to “not think too long about the questions,” with the adolescent sharing, “*maybe it’s just me but that one just seems a little out of place…I don’t think I would think way too long on these questions*” *(female*,* age 16).*

#### Areas of daily life

Nearly three-quarters of participants (n = 16, 73%) found the instructions to Areas of Daily Life section easy to understand. Three adults (out of 12; 25%) and 1 adolescent (out of 10; 10%) found these instructions difficult to understand (2 participants did not provide feedback on the instructions). Specifically, 2 of the adults (17%) and 1 adolescent (10%) reported confusion at being instructed to indicate how often in the last 4 weeks their life had been affected by a swelling episode, regardless of whether they actually had a swelling episode during that time period. Their suggestions for improving these instructions included removing the text in parentheses (i.e., “regardless of whether or not you have actually experienced swelling episodes during that time period”) and/or revising the recall period to “the last time you had an attack/swelling” so that the parenthetical could be eliminated.

*[After reading instructions]…Okay*,* so when I had an attack*,* is that what that means?…so I mean to me*,* that’s—a swelling episode—whether or not you have actually experienced swelling. I don’t know*,* I think it seems to contradict itself. (female*,* age 65)*

*…it was kind of difficult for the first section because it said*,* “When I don’t have swellings*,*” and I like—don’t really have problems other than HAE…it says during the last 4 weeks*,* and I haven’t had swellings and it’s just kinda hard to like answer questions a—like*,* kind of about HAE but not about HAE in like the same way. (female*,* age 12)*

#### Difficulties or problems

Most participants (*n* = 20; 91%) indicated the instructions for the Difficulties or Problems section were easy to understand. One adult considered the instructions confusing because they did not refer specifically to HAE, and another adult was unsure how to answer questions if they had not had an HAE attack in the past 4 weeks. In addition, 3 adults who had reported the instructions to be clear and easy to understand suggested refinements: one felt it would be helpful if the instructions were repeated on each page of the paper version instead of only being presented at the beginning of the section, while another suggested they could be eliminated completely; a third noted it would be helpful if the instructions included an example of a difficulty or problem.

### Response options

All participants (n = 22) were easily able to select a response to each question using the response options provided.

*…very easy*,* um—so I mean they’re the—usually the 5 you see like in the doctor’s office. (female*,* age 65)*

*…these all made complete sense and the fact that there’s 5 of them*,* I think*,* are pretty perfect. (female*,* age 16)*

### Relevance

Nineteen participants (86%) reported that, overall, every item of the AE-QoL applied to their experiences with HAE attacks.

*I don’t think there’s anything that’s not applicable. I think everything is definitely um—relevant to the—to the situation at hand. (male*,* age 20)*

*…the things that it talked about*,* it actually um—related to me a lot*,* um—and I was able to explain how um—it related to me. (male*,* age 17)*

While participants noted that some items within both sections of the AE-QoL were not personally relevant, they did indicate that the concepts measured by these items were still relevant to the overall patient experience of HAE and thus were important to ask.

*I think [the AE-QoL] covers everything. I don’t know if I have too much feedback on the questions other than like I liked ‘em*,* they applied to me…I was kind of on the never or rarely side of things*,* er*,* I think that’s just like specific to my journey with HAE. (male*,* age 26)*

#### Areas of daily life

The following concepts were reported as not relevant by at least 1 participant: Q1. Work (*n* = 4 adolescents out of 10; 40%), Q3. Leisure time (*n* = 1 adolescent out of 10; 10%), and Q5. Eating and drinking (*n* = 2 adults out of 12, 17%; *n* = 3 adolescents out of 10, 30%). Regarding Q1. Work, all 4 adolescents who reported the item as not relevant were not working at the time of their interviews. In many cases, the feedback on relevance was provided in response to a probing question (i.e., “Is this item relevant to your experiences with HAE?”), and not offered spontaneously.

#### Difficulties or problems

Almost three-quarters of participants (n = 16; 73%) reported that 1 or more items in the Difficulties or Problems section were not relevant to them personally in the past 4 weeks, but were relevant to the overall patient experience of HAE and important to ask of people who have HAE (Table 3 provides a breakdown by age group and whether this was reported spontaneously). In many cases, the feedback on relevance was provided in response to a probing question (i.e., “Is this item relevant to your experiences with HAE?”) and not offered spontaneously.

*[In response to the interviewer asking for their thoughts on question 13 “Are you afraid that a swelling episode could occur suddenly?”] I’m not new to this. I’ve been dealing with this for 40 years. So*,* I don’t um—I don’t—I don’t panic. I know the disease. I think that if I was a person that had just been diagnosed or was still learning this*,* then that question would be very relevant because they’re still learning maybe their triggers or they’re still learning um—how to deal with it. (male*,* age 45)*

*…asking*,* like*,* if we felt depressed with…our attacks in*,* like*,* the 4 weeks…[this was not relevant] to me*,* but I feel like it can be…it can apply to*,* like*,* a lot of people simply because mental health is*,* like*,* a big issue nowadays. So I think it is important to include. (female*,* age 17)*

**Table 3:** AE-QoL difficulties and problems items identified as not relevant to participants’ experiences in the past 4 weeks

	Adolescents (*N* = 10)	Adults (*N* = 12)	Reported spontaneously
	*n* (%)	*n* (%)	*n* (%)*
Q6: Do you have difficulty falling asleep?	3 (30)	7 (58)	3 (30)
Q7: Do you wake up during the night?	4 (40)	6 (50)	3 (30)
Q8: Are you tired during the day because you are not sleeping well at night?	4 (40)	7 (58)	1 (9)
Q9: Do you have trouble concentrating?	5 (50)	6 (50)	3 (27)
Q10: Do you feel *depressed*?	7 (70)	5 (42)	4 (33)
Q11: Do you have to limit your choices of food or beverages	3 (30)	5 (42)	2 (25)
Q12: Do the swelling episodes place a burden on you?	3 (30)	1 (8)	1 (25)
Q14: Are you afraid that the frequency of the swelling episodes might increase?	2 (20)	3 (25)	--
Q15: Are you ashamed to go out in public because of the swelling episodes?	1 (10)	4 (33)	4 (80)
Q16: Do the swelling episodes make you embarrassed or self-conscious?	1 (10)	3 (25)	2 (50)
Q17: Are you afraid that the treatment of the swelling episodes could have negative long-term effects for you?	2 (20)	2 (17)	1 (25)

* % based on total n who reported the item as not relevant

Abbreviation: AE-QoL, Angioedema Quality of Life Questionnaire

### Comprehensibility of items

#### Areas of daily life

In general, the items in this section of the AE-QoL were easy for participants to understand, with the exception of Q3. Leisure time. Six participants (1 adult out of 12, 8%; 5 adolescents out of 10, 50%) spontaneously reported that they did not understand the word “leisure;” none were able to correctly guess at its meaning.

*Number 3. How d’you say it? Lei*,* lei—leisurey time—leisure time—luxure time. I can’t read that. What does that mean?…Um—it reminds me of luxury [laughter]. Um—never. [Laughter] Never would be my answer on that one…Cause honestly*,* I don’t know exactly what it’s meaning. (female*,* age 54)*

Interviewer: Can you describe anything um—for me that was confusing or hard to understand when you were answering the questions?

*The third one…I will be honest; I had no idea [laughs]…it’s unfamiliar*,* I haven’t heard that one. (male*,* age 13)*

#### Difficulties or problems

The items in the Difficulties or Problems section were generally easy for all participants to understand. During the think-aloud, participants expressed initial confusion with 6 items (Table 4). However, as they narrated their thought processes, they ultimately understood each item as intended and were able to select a response. Each of these items were further debriefed during the detailed review of the instrument. During this review, participants reported that being reminded of the context of the items as described in the instructions for this section (i.e., associated with your recurrent swelling episodes) helped to ease confusion.

*…do you feel depressed? I would say never for that one…I’m like a little confused. I know you can’t answer this*,* but like do I feel depressed like because of my HAE? Er—I guess that’s kind of the question. Er—I would say never*,* no. I think it’s just kinda like the—the hand I was dealt*,* er—and I have ways to manage it and try not to like let that impact my life too much. So*,* I’ll go with never. (ID 02)*

*…maybe number 9… do you have trouble concentrating?…would that be like after [a swelling] episode*,* during an episode or just overall?…‘cause there’s days I wake up foggy brain…but I don’t think it’s anything about my angioedema*,* I think it’s just me. (ID 20)*

**Table 4:** AE-QoL items reported as unclear during the Think-Aloud activity

	Adolescents (*N* = 10)	Adults (*N* = 12)
	*n* (%)	*n* (%)
Q7: Do you wake up during the night?	--	2 (17)
Q9: Do you have trouble concentrating	--	2 (17)
Q10: Do you feel depressed?	--	1 (8)
Q11: Do you have to limit your choices of food or beverages?	--	1 (8)
Q12: Do the swelling episodes place a burden on you?	2 (20)	--
Q14: Are you afraid the frequency of the swelling episodes might increase?	1 (10)	--

Abbreviation: AE-QoL, Angioedema Quality of Life Questionnaire

### Comprehensiveness

Generally, participants found the AE-QoL to be comprehensive. One reported it to be “all-encompassing” (female, age 65); however, some concepts related to HRQoL were reported as missing from the AE-QoL. These are described below, though it should be noted that these missing concepts did not change the way participants felt about the overall comprehensiveness of the AE-QoL.

Nine adolescents (out of 10; 90%) reported that questions about school-related impacts were missing; 2 (20%) reported this spontaneously and 7 (70%) reported this in response to a probing question. When specifically asked, 8 adolescents (80%) confirmed it would be important to include a question about school. Five adolescents (50%) interpreted Q1. Work as including school, but when questioned specifically, said it would be easier if there was a separate question for school, or if Q1. was “Work/School.”

*I feel like a school section…it could be important to ask ‘cause you could tell what happens with like kids my age*,* how they’re affected more…just like this last section*,* like you could ask like how often you miss school…Or like how often does the swelling affect your study times*,* just like that. (male*,* age 15)*

Three adults (out of 12; 25%) and 2 adolescents (out of 10; 20%) suggested specific impacts on social functioning were missing, including impacts on romantic relationships, impacts on friendships, and how to talk to friends about HAE. One adult (8%) and 1 adolescent (10%) reported that HAE’s impact on the ability to drive was missing; and 2 adults (17%) reported that financial impacts related to HAE were missing. Other impacts noted as missing by 1 participant each (5%) were: daily activities (adult); sports (adolescent); coping with HAE (adult); overall QoL (adult).

*…maybe this is a broad version of the questions that are being asked*,* er—but I think the phrase*,* “quality of life*,*” is very clear and er—universally understood. And so*,* if a question were to read*,* “In the past 4 weeks*,* do you feel like your quality of life has been affected?” And then*,* with the same 5 options. Because saying that you know*,* one attack is depressing or another attack wakes you up during the night*,* isn’t saying that your entire life is miserable. But some people do suffer all the time. That I think is where a question about quality of life would apply. (male*,* age 20)*

## Discussion

Overall, the findings from this study support the content validity of the AE-QoL in adults and adolescents with HAE; this is a first and necessary step in determining that the instrument is a fit-for-purpose measure for capturing symptom-specific impacts (i.e., impacts related to HAE attacks) on HRQoL in this patient population. The results provide evidence that the AE-QoL captures the important aspects of the ways in which HAE impacts adult and adolescent patients’ HRQoL, and that the measure was easy to complete, and, for the most part, easily understood by study participants, including the instructions and the items.

While the content validity of the AE-QoL has been previously evaluated [1, 13, 14], to our knowledge, this is the first study to evaluate its content validity among adolescents. As described earlier, recent guidelines and recommendations [10, 11] for managing HAE state researchers must demonstrate the need to measure changes in patients’ overall functioning rather than only measuring change in HAE symptoms. Evidence that the AE-QoL is content valid for use with adults and adolescents provides researchers with a tool to measure those changes.

Study participants endorsed the brevity of the measure and had no difficulty recalling their experiences over the last 4 weeks. Additionally, they found the response options clear, easy to understand, and appropriate for the questions (items). The general instructions were also clear and easy to understand; however, a small number of participants found the instructions specific to the items asking about Areas of Daily Life to be confusing. Removing the parenthetical text from these instructions could improve understandability for adults and adolescents.

Overall, participants considered the AE-QoL comprehensive and comprehensible; however, a minor revision to Q1., changing it from “Work” to “Work/School” could improve the comprehensiveness of the measure for adolescents. Most adolescent participants noted that the ways HAE impacts school are missing from the measure. While some interpreted Q1. as “school” and answered it this way during the think-aloud, they also indicated it would be important to specifically ask about school and the specific ways in which HAE impacts school attendance and education. Not making this revision to the AE-QoL carries the risk of not capturing HAE impacts on school. In addition, comprehensibility of the AE-QoL could be improved for adolescents with a revision to Q3, changing it from “Leisure time” to “Recreational activities/hobbies,” or to a linguistic equivalent that is familiar to adolescents.

Many of the CD interview findings align with recent studies investigating the content validity of the AE-QoL in adults with HAE [2, 13]. Both Anderson et al. [2] and Vanya et al. [13] found the concepts captured by the AE-QoL to be relevant to adults with HAE. Similar to the current study, participants in the Anderson et al. [2] study noted that impacts on school were missing from the AE-QoL, as were specific impacts related to social functioning.

Similar to the current study, participants in the Vanya et al. [13] study had no difficulty understanding the response options or the recall period. However, the participants in their study also reported no difficulty with the instructions. While participants in the current study easily understood the overall instructions, the instructions specific to the Areas of Daily Life section caused some confusion, which resulted in a suggested revision. It is unclear whether participants in the Vanya et al. [13] study reviewed these secondary instructions together with the general instructions, separately, or not at all.

This study was not without limitations. In particular, most participants had been taking prophylaxis—7 adolescents and 11 adults (82% of the overall sample) reported taking a prophylactic medication at the time of the interview—and reported significant improvements in their symptoms and impacts on HRQoL after initiation of prophylactic treatment. Although the eligibility criteria stipulated participants must have experienced at least 1 HAE attack in the 6 months prior to the interviews, there exists a potential for recall bias in participant accounts of HAE impacts on HRQoL. Additionally, the prophylactic and acute treatments available for HAE are highly efficacious, potentially lessening HRQoL impacts; thus influencing responses about the relevance of concepts measured by the AE-QoL, especially given its 4-week recall period. Finally, since all participants in this study were based in the US, the findings may not be consistent in studies of ex-US populations of patients with HAE.

This study also had several strengths. First, demographically, approximately 30% of the sample self-identified as non-White. Although the findings may not be representative of all individuals with HAE, this study is among the first to include a more diverse sample of patients with HAE, as the samples included in other recent studies have been comprised of participants who were nearly all White (at least 90%) [2, 13]. Additionally, the sample included a balanced representation of age groups among adolescents and self-identified sex within both adults and older adolescents. Second, this study used one-on-one interviews for CD, which aligns with recommendations for conducting qualitative interviews with adolescent populations, as it encourages participants to share their personal experiences and limits the likelihood of social desirability bias [23, 24]. Finally, this study is among the first to use qualitative research methods to evaluate the content validity of the AE-QoL for use with both adults and adolescents.

## Conclusions

This study presents evidence that supports the content validity of the AE-QoL in adult and adolescent patients with HAE. While revisions could be considered prior to using the instrument with samples of adolescent patients with HAE, in general, adolescents and adults with HAE found the measure relevant, comprehensive, and comprehensible. The findings of this study provide valuable insights and detail the first known evaluation of the content validity of the AE-QoL specifically for adolescents with HAE.

## Data Availability

The raw data generated and/or analyzed during the current study (i.e. interview transcripts) are not publicly available due to confidentiality and consent limitations. The interview guide can be made available upon request.

## Footnotes

1 All options for confirming diagnosis aimed to minimize the potential inconvenience of obtaining the documentation and asked participants to, for example, provide an image of prescribed treatment for HAE or a screenshot from their patient portal displaying their diagnosis; the PAG reviewed all documentation and redacted all identifying information, labeling the documents with the participants’ study identification numbers prior to sharing with the study team.

2 Due to challenges recruiting adolescent participants, after extending the recruitment period 3 weeks and evaluating the data to determine new problems were not being reported, a decision was made to end recruitment at 10 adolescent participants instead of the targeted 12.

## Abbreviations

AE: Angioedema
AE-QoL: Angioedema Quality of Life Questionnaire
CD: Cognitive debriefing
HAE: Hereditary angioedema
HRQoL: Health-related quality of life
PAG: Patient advocacy group
PRO: Patient-reported outcome
QoL: Quality of life
RAE: Recurrent angioedema
US: United States
